# Knowledge, attitude, and practice of patients with major depressive disorder on exercise therapy

**DOI:** 10.1186/s12889-024-17821-6

**Published:** 2024-01-29

**Authors:** Junxiang Cheng, Yaoqing Feng, Zhifen Liu, Dandan Zheng, Hong Han, Na Liu, Shasha Liu, Juan Zhao, Xiaole Li, Shifan Han

**Affiliations:** 1 Department of Psychiatry, The First Hospital of Shanxi Medical University, Taiyuan, 030001 China; 2 School of Nursing, Shanxi Medical University, Taiyuan, 030001 China; 3 School of Nursing, Inner Mongolia Medical University, Hohhot, 010110 China; 4 Jinzhou Medical University, Jinzhou, 121000 China; 5 Department of Magnetic Resonance Imaging, Shanxi Bethune Hospital, Taiyuan, 030001 China; 6 Department of Orthopedics, The First Hospital of Shanxi Medical University, Taiyuan, 030001 China; 7 The First Hospital of Shanxi Medical University, Taiyuan, 030001 China

**Keywords:** Knowledge, Attitudes, Practice, Major depressive disorder, Exercise, Education interventions, Cross-sectional study

## Abstract

**Background:**

This study aimed to explore the knowledge, attitude, and practice (KAP) toward exercise therapy of patients with major depressive disorder (MDD).

**Methods:**

This cross-sectional study was conducted at the First Hospital of Shanxi Medical University between April and October 2023 in patients with MDD. A self-designed questionnaire was used to evaluate the KAP (Cronbach’s α = 0.787). The minimum-maximum scores were 2–23 for knowledge, 11–55 for attitude, and 7–35 for practice.

**Results:**

A total of 494 valid questionnaires were analyzed. The mean KAP dimension scores were 15.39 ± 3.34/23 (66.91%), 36.54 ± 19.33/55 (66.44%), and 19.33 ± 5.22/35 (55.23%), indicating poor knowledge, negative attitude, and weak practice. Multivariable logistic regression analysis showed that female (OR = 0.613, 95%CI: 0.376-1.000, *P* = 0.050), urban residence (OR = 0.443, 95%CI: 0.259–0.758, *P* = 0.003), suburban residence (OR = 0.047, 95%CI: 0.016–0.138, *P* < 0.001), higher income (OR = 3.889–7.928, all *P* < 0.001), and unclear self-reported depression level (OR = 0.078, 95%CI: 0.027–0.221, *P* < 0.001) were independently associated with the knowledge scores. Knowledge scores (OR = 1.102, 95%CI: 1.022–1.188, *P* = 0.011), female gender (OR = 0.437, 95%CI: 0.246–0.776, *P* = 0.005), city (OR = 0.410, 95%CI: 0.226–0.744, *P* = 0.003), married (OR = 3.577, 95%CI: 1.751–7.650, *P* < 0.001), higher income (OR = 0.065–0.392, both *P* < 0.050), depressive trend (OR = 2.640, 95%CI: 1.110–6.278, *P* = 0.028), high depression score level (OR = 0.176, 95%CI: 0.104-0.300, *P* < 0.001), and unclear self-reported depression score (OR = 0.023, 95%CI: 0.007–0.076, *P* < 0.001) were independently associated with the attitude scores. Finally, knowledge scores (OR = 1.130, 95%CI: 1.051–1.215, *P* = 0.001), attitude scores (OR = 1.199, 95%CI: 1.124–1.280, *P* < 0.001), and city (OR = 0.583, 95%CI: 0.352–0.965, *P* = 0.036) were independently associated with the practice scores. The structural equation modeling analysis showed that knowledge, but not attitude (β = 0.103, *P* = 0.092) or practice (β = 0.034, *P* = 0.603), influenced the depression level (β=-0.074, *P* < 0.001); attitude influenced practice (β = 0.369, *P* < 0.001).

**Conclusion:**

The KAP toward exercise among MDD patients is poor in Shanxi. Females, people living in urban or suburban areas, with lower income, and self-reported unclear depression levels should be targeted by education interventions.

## Background

Major depressive disorder (MDD) is a common and well-researched type of depressive disorder characterized by persistent low mood, lack of positive affect, and loss of interest in usually pleasurable activities (anhedonia) that is different from the patient’s usual self and causes significant distress or impairment for ≥ 2 weeks [1–3]. MDD is a leading cause of mental health-related disease burden and disability worldwide, affecting an estimated 280 million people [3, 4]. The reported risk factors for MDD include a family or personal history of major depression and/or substance abuse, chronic medical illness, alcohol and substance use, stressful life events including loss (including bereavement or divorce), major life changes such as job change or financial difficulty, domestic abuse or violence, female sex, low income and unemployment, and disability [2, 3]. The prognosis of MDD is variable. It is unremitting in about 15% of patients and recurrent in about 35%, with the risk of recurrence increasing with each additional episode of MDD [2, 3]. It is important to find an effective method to treat and prevent the recurrence of MDD.

Although MDD is often treated with antidepressants and/or psychotherapy [1–3], other treatments have attracted increasing attention in recent years. In particular, physical exercise may be beneficial for depressive symptoms and could have effects comparable to antidepressant drugs [5–7]. Indeed, exercise might improve MDD symptoms compared with no treatment and have similar efficacy as cognitive therapy or sertraline in adults [8]. Various studies revealed that resistance exercise training may reduce depressive symptoms in adults [9], exercise interventions are associated with improved depressive symptoms in patients with neurologic disorders [10], and exercise is associated with improvement in depressive symptoms in sedentary patients with chronic illnesses [11]. The effect appears to be observed in mild-to-moderate MDD [12] but could be less noticeable in severe MDD [13]. Furthermore, low exercise tolerance can be considered a marker of depressive symptoms [14]. Still, exercise appears not to be associated with long-term improvement in MDD in adults beyond the end of intervention [15] and should, therefore, be performed regularly to maintain the benefits.

Even though exercise can be performed in groups or under the supervision of a kinesiologist or a trainer, exercising is based on self-management, influenced by the patient’s knowledge and perception of the benefits and the willingness to exercise [16]. Some studies focused on the knowledge and attitude of MDD patients on exercise intervention therapy [16–18]. A study revealed that general practitioners are generally willing to prescribe exercise to MDD patients [19]. Knowledge, attitude, and practice (KAP) studies provide quantitative and qualitative data about a particular subject in a specific population [20, 21]. They are particularly useful in identifying the knowledge gaps, misconceptions, and misunderstandings that can hinder the optimal performance of the subject and help design educational interventions [20, 21]. Therefore, this study aimed to examine the KAP of patients with MDD on exercise intervention therapy.

## Materials and methods

### Study design and participants

This cross-sectional study was conducted at the First Hospital of Shanxi Medical University between April 2023 and October 2023 and enrolled patients with MDD. The participants were invited to participate in the study if they were outpatients or inpatients diagnosed with MDD by clinicians, in strict accordance with the MDD criteria for ICD-10. The inclusion criteria were (1) 18–65 years of age, (2) met the diagnostic criteria of the International Classification of Diseases, tenth edition (ICD-10) F32 for depressive episodes, and (3) educational level of primary school or above. The exclusion criteria were (1) diagnosis of other physical and psychiatric disorders, (2) difficulty in reading and writing, or (3) severe suicidal ideation and behavior in the last 2 weeks. This study was approved by the First Medical Ethics Committee of Shanxi Medical University. All participants signed the informed consent form before completing the questionnaire.

### Questionnaire

This questionnaire was designed based on the literature [5–11, 22]. The first draft of the questionnaire was revised according to the opinions of five senior experts. A pilot evaluation (35 copies) was performed and showed a Cronbach’s α of 0.787.

The final questionnaire was in Chinese and included four aspects: demographic data (age, marital status, place of residence, educational level, gender, income, type of medical insurance, depression trend, current therapies received, self-reported depression level, and ways to learn about depression), knowledge dimension (depression and its pathogenesis, prevention, clinical symptoms, and management), attitude dimension, and practice dimension. The knowledge dimension included 10 questions. One point was scored for correct answers, and zero points for other answers. Items K5-K7 included several subitems, and one point was scored for correct answers. The total scores of items K5-K7 were 5, 6, and 5 points, respectively. The score range was 2–23 points. The attitude dimension consisted of 11 questions using a 5-point Likert scale from very positive (5 points) to very negative (1 point). The score range was 11–55 points. The practice dimension consisted of 10 questions using a 5-point Likert scale from always (5 points) to never (1 point). The score range was 7–35 points.

The questionnaires were distributed to the participants at the outpatient clinic and wards of the First Hospital of Shanxi Medical University using posters. The participants could scan the QR code to gain access to a description of the study, consent form, and questionnaire. Members of the research team were also available to answer questions via telephone, email, or WeChat. Questionnaires that took < 1 min to complete, with obvious logical errors, or completed using the same option (e.g., all first choices) were considered invalid. In order to avoid repetition, IP restriction was applied, thus making sure that the survey could only be completed once from a single IP address. A total score of each dimension ≥ 70% was defined as adequate knowledge, positive attitude, and proactive practice [23].

### Statistical analysis

The sample size for this study was calculated based on the guideline of having 10 times the number of questionnaire items [24], which, in this study, was 31 independent variables. Therefore, the minimum sample size required was 310 participants. A minimum of 389 participants was required to accommodate a potential 20% non-valid questionnaire rate in survey responses.

Stata 17.0 (Stata Corporation, College Station, TX, USA) was used for statistical analysis. The demographic data and KAP scores of the participants were analyzed descriptively using means ± standard deviations (SD) for continuous data and n (%) for categorical data. Continuous data with a normal distribution were analyzed using Student’s t-test (comparison of two groups) or ANOVA (comparison of three or more groups). Continuous data with a skewed distribution were analyzed using the Wilcoxon-Mann-Whitney test (comparison of two groups) or the Kruskal-Wallis analysis of variance (comparison of three or more groups). The categorical data were analyzed using the chi-squared test. Pearson correlation analysis was used to evaluate the correlation among KAP dimensions. Multivariable regression analyses were carried out with the attitude and practice scores as dependent variables to analyze the relationship between demographic data or knowledge or attitude and practice. The mean score of each dimension was used as the cutoff point [25, 26]. Variables with *P* < 0.05 in univariable analyses were included in the multivariable analyses. Structural equation modeling (SEM) was used to examine the relationships among the KAP dimensions and other factors. Two-sided P-values < 0.05 were considered statistically significant.

## Results

### Characteristics of the participants

A total of 522 questionnaires were collected, but 494 were included in the analyses after excluding 28 invalid questionnaires. Most participants were female (68.22%), ages 18–27 (50.61%), living in city (59.51%), unmarried (55.87%), with college or bachelor’s degree or above (40.69%), earning 2000–4999 per month (33.60%), with medical insurance (100%), with depressive tendency (87.45%), and with a depression level > 73 (51.42%) **(**Table 1**).**

**Table 1:** Characteristics of the participants

Variables	n (%)	Knowledge scores		Attitude scores		Practice scores	
		Mean ± SD	P	Mean ± SD	P	Mean ± SD	P
Total scores	494	15.39 ± 3.34		36.54 ± 19.33		19.33 ± 5.22	
Gender			0.802		< 0.001		< 0.001
Male	157 (31.78)	15.34 ± 3.16		37.67 ± 3.42		21.52 ± 5.43	
Female	337 (68.22)	15.42 ± 3.42		36.02 ± 4.92		18.31 ± 4.80	
Age			0.055		< 0.001		< 0.001
[18, 27]	250 (50.61)	15.11 ± 3.49		35.53 ± 4.90		18.15 ± 4.81	
(27, 72)	244 (49.39)	15.68 ± 5.15		37.58 ± 3.93		20.54 ± 5.36	
Residence			< 0.001		< 0.001		< 0.001
Country	167 (33.81)	14.91 ± 3.38		38.16 ± 4.73		21.04 ± 5.69	
City	294 (59.51)	15.86 ± 3.27		35.86 ± 4.15		18.33 ± 4.30	
Suburbs	33 (6.68)	13.67 ± 2.77		34.45 ± 4.87		19.58 ± 7.64	
Marital status			0.394		< 0.001		< 0.001
Unmarried	276 (55.87)	15.32 ± 3.28		35.60 ± 4.97		18.49 ± 4.99	
Married	206 (41.70)	15.56 ± 3.44		37.64 ± 3.64		20.50 ± 5.29	
Divorced or widowed	12 (2.43)	14.33 ± 2.87		39.42 ± 3.60		18.58 ± 5.85	
Education			0.073		< 0.001		0.003
Junior high and lower	108 (21.86)	14.76 ± 3.50		37.91 ± 4.12		20.44 ± 7.17	
High school/technical secondary school	185 (37.45)	15.66 ± 2.71		35.07 ± 4.57		19.65 ± 4.82	
College/bachelor or above	201 (40.69)	15.49 ± 3.71		37.16 ± 4.42		18.44 ± 4.10	
Monthly per capita income, RMB			< 0.001		< 0.001		< 0.001
< 2000	91 (18.42)	13.29 ± 3.51		38.31 ± 4.08		21.68 ± 6.73	
2000–4999	166 (33.60)	15.60 ± 3.19		36.92 ± 4.86		18.11 ± 5.77	
5000–9999	165 (33.40)	15.73 ± 3.42		36.44 ± 3.38		19.88 ± 3.67	
≥ 10,000	72 (14.57)	16.81 ± 1.72		33.68 ± 5.40		17.90 ± 3.21	
Medical insurance			0.088		0.735		0.853
Single social health insurance	419 (84.82)	15.50 ± 3.20		36.51 ± 4.27		19.35 ± 5.31	
Both social medical insurance and commercial medical insurance	75 (15.18)	14.79 ± 4.00		36.71 ± 5.96		19.23 ± 4.76	
Depression			< 0.001		< 0.001		0.023
Yes	432 (87.45)	15.69 ± 3.25		36.89 ± 4.54		19.53 ± 5.19	
No or unclear	62 (12.55)	13.35 ± 3.27		34.13 ± 3.92		17.92 ± 5.30	
Current therapies (multiple options)			-		-		-
Drug	494	-		-		-	
Psychological counseling	243	-		-		-	
Exercise therapy	56	-		-		-	
Traditional Chinese medicine	4	-		-		-	
Self-reported depression level			< 0.001		< 0.001		< 0.001
≤73	200 (40.49)	15.97 ± 2.67		38.75 ± 3.64		21.29 ± 5.57	
> 73	254 (51.42)	15.65 ± 3.28		35.22 ± 4.62		18.09 ± 4.56	
Unclear	40 (8.10)	10.90 ± 3.43		33.88 ± 3.61		17.45 ± 4.26	
Ways to learn about depression (multiple options)			-		-		-
Healthcare workers	338	-		-		-	
Health Book	143	-		-		-	
Family and friends	74	-		-		-	
Television networks	434	-		-		-	
Others	37	-		-		-	

### Knowledge

The mean knowledge score was 15.39 ± 3.34 on a maximum of 23 (66.91%). Higher knowledge scores were observed in urban residents (*P* < 0.001), with higher income (*P* < 0.001), with a depressive trend (*P* < 0.001), and with lower depression scores (*P* < 0.001) (Table 1). The knowledge item with the highest score was K1 (96.96%; “Depression is a mental illness”), while the item with the lowest score was K3 (5.26%; “Most depression treatments focus on preventing its recurrence”) **(**Table 2**).**

**Table 2:** Knowledge dimension

Knowledge	Correct, n (%)
K1. Depression is a mental illness.	479 (96.96)
K2. Depression can be cured.	356 (72.06)
K3. Most depression treatments focus on preventing its recurrence.	26 (5.26)
K4. Antidepressants have no side effects	371 (75.1)
K5. Common symptoms of depression	
K5.1. Low mood	493 (99.8)
K5.2. Slow thinking	494 (100)
K5.3 Insomnia or drowsiness	483 (97.77)
K5.4. Cognitive dysfunction (memory loss, etc.)	469 (94.94)
K5.5. Shame and self-harm	466 (94.33)
K5.6. Miscellaneous	143 (28.95)
K6. Ways to relieve and treat depression.	
K6.1. Psychological counseling	480 (97.17)
K6.2. Active exercise	399 (80.77)
K6.3. Drug	467 (94.53)
K6.4. Travel	336 (68.02)
K6.5. Confide in friends and family	330 (66.8)
K6.6. Develop personal hobbies that are beneficial to the body and mind	376 (76.11)
K7. Factors that cause depression	
K7.1. Inheritance	202 (40.89)
K7.2. Personal characteristics	494 (100)
K7.3. Social environment and living environment	478 (96.76)
K7.4. Chronic diseases	116 (23.48)
K7.5. Bad living habits	227 (45.95)
K8. Proper exercise can improve depression.	380 (76.92)
K9. Is depression better to improve through exercise or drug	174 (35.22)
K10. Exercise can be used as an alternative to speech therapy or drug	211 (42.71)

### Attitudes

The mean attitude score was 36.54 ± 19.33 on a maximum of 55 (66.44%). Higher attitude scores were observed in males (*P* < 0.001), 27–72 age group (*P* < 0.001), in rural areas (*P* < 0.001), divorced/widowed (*P* < 0.001), with junior high or lower education (*P* < 0.001), with lower income (*P* < 0.001), with a depressive trend (*P* < 0.001), and with lower depression scores (*P* < 0.001) **(**Table 1**)**. Table 3 presents the distribution of the answers to the attitude items.

**Table 3:** Attitude dimension

	Strongly agree (n, %)	Agree (n, %)	Neutrality (n, %)	Disagree (n, %)	Strongly disagree (n, %)
A1. You agree that exercise makes you physically and mentally happy and healthier.	57 (11.54)	230 (46.56)	164 (33.2)	7 (1.42)	36 (7.29)
A2. You think exercise is important for your body.	131 (26.52)	226 (45.75)	93 (18.83)	14 (2.83)	30 (6.07)
A3. You think exercise improves depression.	47 (9.51)	252 (51.01)	149 (30.16)	17 (3.44)	29 (5.87)
A4. You think their depression is nothing that does not require treatment.	0 (0)	23 (4.66)	62 (12.55)	172 (34.82)	237 (47.98)
A5. You agree that sports should be popularized in everybody’s life, the installation of related facilities should be increased, and long-term habits should be maintained.	167 (33.81)	206 (41.7)	82 (16.6)	31 (6.28)	8 (1.62)
A6. If exercise therapy can help you improve depression, you are willing to try it.	93 (18.83)	250 (50.61)	131 (26.52)	14 (2.83)	6 (1.21)
A7. You don’t think you need additional treatment after you stick to exercise.	25 (5.06)	29 (5.87)	103 (20.85)	214 (43.32)	123 (24.9)
A8. You believe that if depression is controlled after regular exercise, exercise therapy is still needed.	81 (16.4)	230 (46.56)	159 (32.19)	7 (1.42)	17 (3.44)
A9. If you have a professional medical staff to guide the exercise, are you willing to participate?	90 (18.22)	176 (35.63)	199 (40.28)	14 (2.83)	15 (3.04)
A10. If your healthcare provider has developed an exercise plan for you, are you willing to implement it?	109 (22.06)	225 (45.55)	131 (26.52)	29 (5.87)	0 (0)
	A	B	C	D	E
A11	225 (45.55)	15 (3.04)	30 (6.07)	209 (42.31)	15 (3.04)

### Practice

The mean practice score was 19.33 ± 5.22 on a maximum of 35 (55.23%). Higher practice scores were observed in males (*P* < 0.001), 27–72 age group (*P* < 0.001), in rural areas (*P* < 0.001), married (*P* < 0.001), with junior high or lower education (*P* = 0.003), with lower income (*P* < 0.001), with a depressive trend (*P* = 0.023), and with lower depression scores (*P* < 0.001) **(**Table 1**)**. Table 4 presents the distribution of the answers to the practice items.

**Table 4:** Practice dimension

	Always (n, %)	Usually (n, %)	Sometimes (n, %)	Occasionally (n, %)	Never (n, %)
P1. Do you participate in exercise after a busy day’s work?	15 (3.04)	45 (9.11)	136 (27.53)	151 (30.57)	147 (29.76)
P2. Is your mood significantly better after exercising?	15 (3.04)	91 (18.42)	148 (29.96)	195 (39.47)	45 (9.11)
P3. Do you take exercise as a long-term habit or the first solution to get rid of bad moods?	59 (11.94)	46 (9.31)	104 (21.05)	165 (33.4)	120 (24.29)
P4. Existing studies have shown that exercise can improve depression to a certain extent and stabilize the psychological state of depressed patients. Will you promote this therapy to help more depressed patients?	130 (26.32)	121 (24.49)	134 (27.13)	90 (18.22)	19 (3.85)
P5. Do you vent your emotions with exercise?	30 (6.07)	30 (6.07)	152 (30.77)	105 (21.26)	177 (35.83)
P6. Do you monitor your heart rate during exercise?	30 (6.07)	45 (9.11)	58 (11.74)	75 (15.18)	286 (57.89)
P7. Do you have regular exercise?	0 (0)	45 (9.11)	225 (45.55)	222 (44.94)	2 (0.4)

### Correlations

The knowledge scores were not correlated to the attitude (*r* = 0.076, *P* = 0.093) or practice (*r* = 0.086, *P* = 0.057) scores. The attitude scores were correlated to the practice scores (*r* = 0.394, *P* < 0.001).

### Multivariable analyses

Female gender (OR = 0.613, 95%CI: 0.376-1.000, *P* = 0.050), urban residence (OR = 0.443, 95%CI: 0.259–0.758, *P* = 0.003), suburban residence (OR = 0.047, 95%CI: 0.016–0.138, *P* < 0.001), income 2000–4999 RMB (OR = 3.889, 95%CI: 1.959–7.721, *P* < 0.001), income 5000–9999 RMB (OR = 5.802, 95%CI: 2.875–11.709, *P* < 0.001), income ≥ 10,000 RMB (OR = 7.928, 95%CI: 3.419–18.386, *P* < 0.001), and unclear self-reported depression level (OR = 0.078, 95%CI: 0.027–0.221, *P* < 0.001 were independently associated with the knowledge scores **(**Table 5**)**.

**Table 5:** Univariable and multivariable analyses of knowledge, attitude, and practice

Variables	Univariable analysis		Multivariable analysis	
	OR (95%CI)	P	OR (95%CI)	P
**Knowledge**				
Gender				
Male	ref		ref	
Female	0.596 (0.401 0.885)	0.010	0.613 (0.376 1.000)	0.050
Age				
[18, 27]	ref		ref	
(27, 72)	1.613 (1.124 2.314)	0.009	1.166 (0.760 1.789)	0.482
Residence				
Country	ref		ref	
City	1.166 (0.790 1.720)	0.440	0.443 (0.259 0.758)	0.003
Outskirts	0.123 (0.045 0.334)	< 0.001	0.047 (0.016 0.138)	< 0.001
Marital status				
Unmarried	ref			
Married	1.434 (0.990 2.077)	0.057		
Divorced or widowed	0.402 (0.118 1.366)	0.144		
Education				
Junior high and lower				
High school/technical secondary school	1.147 (0.710 1.853)	0.574		
College/bachelor or above	1.185 (0.739 1.902)	0.481		
Monthly per capita income, RMB				
< 2000	ref		ref	
2000–4999	2.406 (1.415 4.091)	0.001	3.889 (1.959 7.721)	< 0.001
5000–9999	3.871 (2.253 6.649)	< 0.001	5.802 (2.875 11.709)	< 0.001
≥ 10,000	6.774 (3.348 13.706)	< 0.001	7.928 (3.419 18.386)	< 0.001
Medical insurance				
Single social health insurance	ref			
Both social medical insurance and commercial medical insurance	0.732 (0.447 1.199)	0.216		
Depression				
Yes	2.512 (1.455 4.339)	0.001	1.333 (0.670 2651)	0.413
No or unclear	ref		ref	
Self-reported depression level				
≤73	ref		ref	
> 73	0.829 (0.565 1.218)	0.340	0.715 (0.445 1.149)	0.166
Unclear	0.077 (0.029 0.205)	< 0.001	0.078 (0.027 0.221)	< 0.001
**Attitude**				
Knowledge scores	1.101 (1.042 1.162)	0.001	1.102 (1.022 1.188)	0.011
Gender				
Male	ref		ref	
Female	0.470 (0.315 0.702)	< 0.001	0.437 (0.246 0.776)	0.005
Age				
[18, 27]	ref		ref	
(27, 72)	2.505 (1.736 3.616)	< 0.001	0.969 (0.504 1.863)	0.924
Residence				
Country	ref		ref	
City	0.512 (0.344 0.761)	0.001	0.410 (0.226 0.744)	0.003
Outskirts	0.573 (0.269 1.224)	0.151	0.401 (0.148 1.087)	0.072
Marital status				
Unmarried	ref		ref	
Married	2.608 (1.784 3.815)	< 0.001	3.577 (1.751 7.65)	< 0.001
Divorced or widowed	3.369 (0.893 12.712)	0.073	4.442 (0.874 22.574)	0.072
Education				
Junior high and lower	ref		ref	
High school/technical secondary school	0.562 (0.347 0.910)	0.019	0.941 (0.450 1.965)	0.871
College/bachelor or above	1.294 (0.798 2.098)	0.296	1.606 (0.791 3.261)	0.190
Monthly per capita income, RMB				
< 2000	ref		ref	
2000–4999	0.562 (0.325 0.974)	0.040	0.508 (0.208 1.242)	0.138
5000–9999	0.543 (0.313 0.941)	0.029	0.392 (0.159 0.962)	0.041
≥ 10,000	0.226 (0.117 0.438)	< 0.001	0.065 (0.022 0.189)	< 0.001
Medical insurance				
Single social health insurance	ref			
Both social medical insurance and commercial medical insurance	0.828 (0.505 1.356)	0.453		
Depression				
Yes	2.775 (1.593 4.833)	< 0.001	2.640 (1.110 6.278)	0.028
No or unclear	ref		ref	
Self-reported depression level				
≤73	ref		ref	
> 73	0.116 (0.073 0.184)	< 0.001	0.176 (0.104 0.300)	< 0.001
Unclear	0.049 (0.021 0.114)	< 0.001	0.023 (0.007 0.076)	< 0.001
**Practice**				
Knowledge scores	1.123 (1.061 1.189)	< 0.001	1.130 (1.051 1.215)	0.001
Attitude scores	1.256 (1.188 1.328)	< 0.001	1.199 (1.124 1.280)	< 0.001
Gender				
Male	ref			
Female	0.710 (0.486 1.039)	0.078		
Age				
[18, 27]	ref		ref	
(27, 72)	1.714 (1.198 2.452)	0.003	1.318 (0.853 2.035)	0.214
Residence				
Country	ref		ref	
City	0.494 (0.336 0.727)	< 0.001	0.583 (0.352 0.965)	0.036
Outskirts	0.866 (0.410 1.829)	0.706	1.616 (0.633 4.125)	0.315
Marital status				
Unmarried	ref			
Married	1.233 (0.858 1.773)	0.258		
Divorced or widowed	0.971 (0.301 3.134)	0.960		
Education				
Junior high and lower	ref			
High school/technical secondary school	1.312 (0.814 2.114)	0.265		
College/bachelor or above	0.858 (0.534 1.378)	0.527		
Monthly per capita income, RMB				
< 2000	ref		ref	
2000–4999	0.842 (0.505 1.406)	0.512	1.004 (0.499 2.017)	0.992
5000–9999	1.060 (0.635 1.769)	0.823	1.439 (0.718 2.883)	0.305
≥ 10,000	0.292 (0.146 0.583)	< 0.001	0.441 (0.180 1.082)	0.074
Medical insurance				
Single social health insurance	ref			
Both social medical insurance and commercial medical insurance	0.914 (0.557 1.502)	0.724		
Depression				
Yes	2.147 (1.202 3.835)	0.010	0.990 (0.495 1.983)	0.978
No or unclear	ref		ref	
Self-reported depression level				
≤73	ref			
> 73	0.338 (0.230 0.496)	< 0.001	0.650 (0.414 1.021)	0.062
Unclear	0.157 (0.069 0.357)	< 0.001	0.514 (0.183 1.442)	0.206

The knowledge scores (OR = 1.102, 95%CI: 1.022–1.188, *P* = 0.011), female gender (OR = 0.437, 95%CI: 0.246–0.776, *P* = 0.005), city (OR = 0.410, 95%CI: 0.226–0.744, *P* = 0.003), married (OR = 3.577, 95%CI: 1.751–7.650, *P* < 0.001), income 5000–9999 RMB (OR = 0.392, 95%CI: 0.159–0.962, *P* = 0.041), income ≥ 10,000 RMB (OR = 0.065, 95%CI: 0.022–0.189, *P* < 0.001), depressive trend (OR = 2.640, 95%CI: 1.110–6.278, *P* = 0.028), high depression score level (OR = 0.176, 95%CI: 0.104-0.300, *P* < 0.001), and self-reported unclear depression level (OR = 0.023, 95%CI: 0.007–0.076, *P* < 0.001) were independently associated with the attitude scores **(**Table 5**)**.

The knowledge scores (OR = 1.130, 95%CI: 1.051–1.215, *P* = 0.001), attitude scores (OR = 1.199, 95%CI: 1.124–1.280, *P* < 0.001), and city (OR = 0.583, 95%CI: 0.352–0.965, *P* = 0.036) were independently associated with the practice scores **(**Table 5**)**.

### Structural equation modeling

**Fig. 1:**
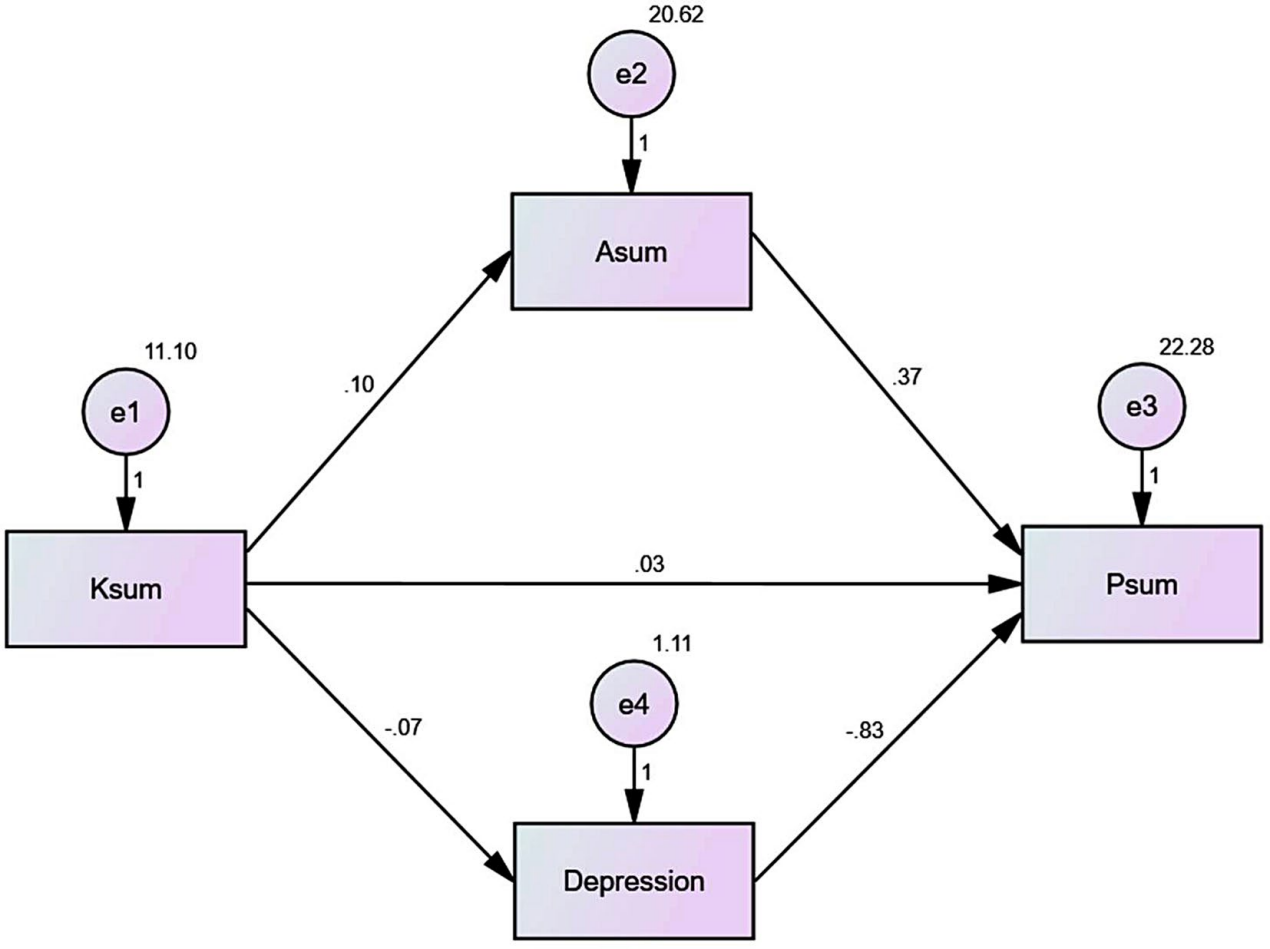
Structural equation modeling

The SEM showed that the knowledge influenced the depression level (β=-0.074, *P* < 0.001) but not the attitude (β = 0.103, *P* = 0.092) or practice (β = 0.034, *P* = 0.603). The attitude influenced the practice (β = 0.369, *P* < 0.001). The depression level influenced practice (β=-0.827, *P* < 0.001) (Table 6; Fig. 1).

**Table 6:** Parameters of the structural equation modeling

Relationship			Estimate	P
Depression level	<---	K	-0.074	< 0.001
A	<---	K	0.103	0.092
P	<---	Depression level	-0.827	< 0.001
P	<---	A	0.369	< 0.001
P	<---	K	0.034	0.603

## Discussion

Patients with MDD in Shanxi have poor knowledge about exercise as a way to treat MDD, they have a negative attitude toward exercise, and they do not exercise. Being female, living in urban or suburban, with a lower income, and unclear self-reported depression levels have negative impacts on the KAP about exercise as a way to treat MDD. The results may help optimize the performance of exercise therapy in depressive patients.

Most participants were female, consistent with the higher prevalence of MDD among females [2, 3]. Most participants were unmarried and with a middle-low income, consistent with the association of MDD with low income and relationship difficulties [2, 3]. Exercise is recognized to improve the symptoms of MDD and have an impact on the risk of recurrence [10, 11, 22], but exercise must be practiced regularly to have a lasting effect [15]. Exercising requires time, and patient self-management is crucial in the quantity and quality of exercise performed by an individual [27, 28]. Hence, a proper knowledge of the potential benefits of exercise on physical and mental health could improve the attitudes and practice toward exercise among patients with MDD. In the present study, the participants had a generally good grasp of the symptoms, risk factors, and management of MDD but a lower knowledge of the prognosis of MDD, the impact of treatments on recurrence, drug side effects, and the impact of exercise on managing MDD. Patient education interventions should be designed and implemented to improve these points. Lower knowledge was also observed in females, people living in urban or suburban, low income, and an unclear self-reported depression level. Socioeconomic status is often associated with better health literacy [29], as observed here. On the other hand, living in a city can be associated with a more stressful life and less access to parks and sports facilities, leading to a lower willingness to gain knowledge on exercise with fewer opportunities to practice it. Not knowing their depressive status can suggest disinterest in one’s health and related knowledge. Females usually have a higher health literacy than males [30], but the higher rate and severity of MDD in females than in males could bias the association of knowledge. The SEM analysis indeed suggests that the depression level influenced knowledge.

Knowledge was associated with attitude and practice, and attitude was associated with practice, as supported by the KAP theory that stipulates that knowledge is the basis for changes in habits while attitudes are the driving force of the changes [20, 21]. The low attitude and practice observed in the city support the above theory about poor access to sports and activity facilities. The inverse relationship between income and attitudes could be related to the workload and responsibilities and less time and willingness to exercise. The SEM analysis also showed that the depression level affected practice, probably because of a lower willingness to exercise.

Previous data on the KAP toward exercise in patients with MDD are scarce. Doyle et al. [16] reported that 31%, 29%, and 26% of the participants in the United Emirates did not believe that exercise could improve well-being, manage depression, and manage stress/anxiety, respectively. However, a study in Portugal showed that 87.8% of the participants believed that exercise could reduce depressive symptoms [17]. A study in South Korea showed that an exercise program could improve the KAP about exercise and MDD [18]. Hence, efforts should be taken to improve the KAP toward exercise and MDD, especially among patients with MDD.

This study has limitations. It was performed at a single hospital, limiting the sample size and the geographical source of the participants and, hence, generalizability. The study was advertised on posters, and the participants had to scan the QR code, leading to volunteer and nonresponse biases. The questionnaire was self-designed by the investigators and might be biased by local practices, guidelines, and policies. Still, KAP questionnaires are usually developed by local investigators based on local practices, policies, and customs. Otherwise, the questionnaire might miss or misinterpret local realities. It limits the generalizability of the results and the exportability of the questionnaire. A cross-sectional KAP study cannot provide data about cause-to-effect relationships and provide a snapshot of the situation at a precise point in time. Nevertheless, the results could eventually be used as a historical baseline to evaluate the impact of future interventions. The study included several self-reported variables, which are susceptible to recall bias. A SEM analysis was used to examine the relationships among KAP dimensions and other variables, but it is an artificial construct based on predefined hypotheses, and it cannot be used to determine causality with certainty. Finally, all KAP studies are at risk of the social desirability bias, in which some participants can answer what they should do (based on a socially acceptable opinion or behavior) instead of what they really do [31, 32]. Additional studies are necessary to refine our understanding of the KAP toward exercise among depressive patients nationwide.

## Conclusions

In conclusion, patients with MDD in Shanxi have poor knowledge about exercise as a way to treat MDD, they have a negative attitude toward exercise, and they do not exercise. Being female, living in urban or suburban, with a lower income, and unclear self-reported depression levels have negative impacts on the KAP about exercise as a way to treat depression. Future studies should examine the KAP toward exercise in relation to the actual level of physical activity and the depression outcomes in depressive patients.

## Data Availability

All data generated or analyzed during this study are included in this published article.

## Abbreviations

KAP: knowledge, attitude, and practice
MDD: Major depressive disorder
SEM: Structural equation modeling
